# Cryoprotectant Toxicity: Facts, Issues, and Questions

**DOI:** 10.1089/rej.2014.1656

**Published:** 2015-10-01

**Authors:** Benjamin P. Best

**Affiliations:** Life Extension Foundation, Fort Lauderdale, Florida.

## Abstract

High levels of penetrating cryoprotectants (CPAs) can eliminate ice formation during cryopreservation of cells, tissues, and organs to cryogenic temperatures. But CPAs become increasingly toxic as concentration increases. Many strategies have been attempted to overcome the problem of eliminating ice while minimizing toxicity, such as attempting to optimize cooling and warming rates, or attempting to optimize time of adding individual CPAs during cooling. Because strategies currently used are not adequate, CPA toxicity remains the greatest obstacle to cryopreservation. CPA toxicity stands in the way of cryogenic cryopreservation of human organs, a procedure that has the potential to save many lives. This review attempts to describe what is known about CPA toxicity, theories of CPA toxicity, and strategies to reduce CPA toxicity. Critical analysis and suggestions are also included.

## Introduction

The availability of transplantable organs could considerably postpone 30% of all deaths in the United States. But the demand for transplantable organs greatly exceeds the supply. Reversible cryopreservation of transplantable organs at cryogenic temperatures could substantially increase their availability.^1^

Cryoprotective agents (CPAs) are used to eliminate ice formation when cooling organs to cryogenic temperatures.^2^ Organs could be cryopreserved without ice formation if there were no limit to the amount of CPA that could be used, but toxicity of CPAs limits the amount that can be used.^3^ CPA toxicity has been described as the major impediment to cryopreservation by vitrification.^2,4^ Understanding the mechanisms of CPA toxicity to know how to reduce CPA toxicity could be the means to successful organ cryopreservation.

This review will attempt to present an overview of CPA toxicity on the broadest possible level. Many, if not most, cryopreservation researchers seem to have the view that CPA toxicity follows different rules for different cells, tissues, or organisms.^5^ Yet all cells, tissues, and organisms are composed of similar cellular components and macromolecules. Understanding the reasons for differing toxicities in different biological environments can lead to understanding the mechanisms of CPA toxicity. If erythrocytes or embryos of one species show very different CPA toxicities from erythrocytes or embryos of another species, understanding the reasons for those differences should provide insight into toxicity mechanisms. This review does not presume to explain the many puzzling differences seen in cryopreservation of different biological systems with different CPAs, but rather attempts to present results seen empirically in the hope of serving as an impetus for others to discover explanations.

Many of the differences in the results of CPA toxicity research arise because of different experimental conditions, such as temperature, CPA concentration, CPA exposure time, CPA carrier solution, and type of toxicity assays (viability assay). CPAs may be deemed toxic if cell membranes are breached or damaged, if enzyme function is impaired, if cell or embryo development is diminished, if sperm motility is impaired, if mitochondrial function is reduced, or if DNA, protein, or other macromolecules are damaged. Some effects deemed to be due to CPA toxicity may actually be due to osmotic shock, oxidative stress, chilling injury, or other causes of damage.

Toxicity can be specific to a particular CPA (specific toxicity) or toxicity that is a consequence of being a CPA (non-specific toxicity).^6–8^ CPAs are believed to prevent ice formation by interfering with hydrogen bonding between water molecules,^9^ and this effect has been proposed to cause non-specific toxicity.^8^

The focus of this review will be on widely used CPAs that cross cell membranes (“penetrating CPAs”), namely, ethylene glycol (EG), propylene glycol (PG; 1,2-propanediol), dimethylsulfoxide (DMSO), glycerol (GLY), formamide (FMD), methanol (METH), and butanediol (BD; 2,3-butanediol). The review begins with a description of specific CPA toxicities and specific forms of damage. Some comparative CPA studies follow. The final sections deal with theories of CPA toxicity or strategies to achieve CPA toxicity neutralization.

## CPA-Specific Toxicities

Although some of the specific CPA toxicities discussed only occur at high temperature or to particular cells or organs, it is possible that awareness of these effects could shed light on injuries associated with these CPAs during their use for cryopreservation.

EG is metabolized (primarily in the liver) by alcohol dehydrogenase to glycoaldehyde and then by aldehyde dehydrogenase to produce glycolic acid, which can result in metabolic acidosis. Glycolic acid can be further metabolized to oxalic acid, which precipitates with calcium to form calcium oxalate crystals in many tissues, notably the kidney.^10–13^ Metabolism of EG to an extent that elicits clinically significant symptoms can take hours at body temperature. Because of the time required and because metabolism is mainly in the liver, this form of toxicity is probably not relevant to rapid hypothermic procedures used to cryopreserve organs, tissues, and cells. Independent of the effects of calcium oxalate, EG can cause gastrointestinal irritation and pulmonary edema^14^ as well as widespread inflammation of the lungs.^15^

PG has few systemic toxic effects as evidenced by the fact that it has been safely used in many food products. PG has been used as an antidote for EG poisoning.^16^ Nonetheless, PG often exhibits toxicity when used as a CPA. For example, PG in excess of 2.5 M has been shown to impair the developmental potential of mouse zygotes by decreasing intracellular pH.^17^

Cryopreservation of spermatozoa by GLY was a major breakthrough for cryobiology.^18^ Nonetheless, some injuries are evident.^19^ Systemically, 10 mL of 50% GLY per kilogram induces renal failure in rats through inflammation, oxidative stress, and apoptosis.^20^ All of these processes are facilitated by caspases.^21^ GLY depletes reduced glutathione in the kidney, leading to oxidative stress.^22^ In stallion spermatozoa, GLY in concentrations over 1.5% polymerizes the actin cytoskeleton, a phenomenon unrelated to osmolality.^23^ Freezing human sperm with 15% GLY is equally likely to damage sperm morphology, mitochondria, and viability, but reduction in motility was shown to correlate with reduction in mitochondrial function.^24^ GLY is reportedly much more toxic than other CPAs for flounder embryos^25^ and *Escherichia coli* bacteria.^26^

FMD is a highly corrosive amide that has been used for manufacturing plastics. Inhalation of large amounts of FMD can require medical attention due to kidney and blood cell injury,^27^ although the molecular mechanisms have not been carefully studied. As with water, FMD molecules can form four intermolecular hydrogen bonds, and therefore pure FMD solution will form networks as water does.^28^ The dipole moment of the FMD molecule is roughly twice that of a water molecule.^29^ FMD hydrogen bonding to water is about 10– 20% stronger than hydrogen bonding between water molecules.^30,31^ Mixed with water, FMD molecules more strongly self-associate than associate with water molecules, which may explain why FMD cannot vitrify in an aqueous solution without assistance from other CPAs.^4^ A FMD molecule hydrogen-bonds to another FMD molecule with a strength of −5.51 kcal/mol, but a chain of 12 FMD molecules can reach hydrogen-bonding strength of −13.66 kcal/mol.^32^ These hydrogen-bonding strengths are greater than the hydrogen bond strength between water molecules (−4.46 kcal/mol).^33^ FMD can denature DNA, an effect believed to be due to displacement of hydrating water.^30^

METH is metabolized to formaldehyde and then to formic acid by alcohol dehydrogenase,^34^ which can cause metabolic acidosis, cardiovascular instability, and blindness by destruction of the optic nerve.^35,36^ METH is more polar than ethanol, and thus cannot penetrate through the lipid chain regions of cell membranes as ethanol can.^37^ Nonetheless, because of its small size, methanol is able to cross cell membranes through pores.^38^ In zebrafish ovarian follicles, cryopreservation with METH showed a dose-dependent reduction in five mitochondrial function measures—membrane potential, mitochondrial distribution, mitochondrial DNA copy number, adenosine triphosphate (ATP) levels, and adenosine diphosphate (ADP/ATP ratios.^39^ A study of fish oocytes found that methanol concentrations above 6 M (but not below) resulted in protein damage or proteolysis.^40^

Butanediol has four stable structural isomers, only two of which (1,3-butanediol and 2,3-butanediol, *i.e*., BD) have been used as CPAs. The CPA properties of 1,3-butanediol are reportedly similar to those of PG, while being slightly more toxic than PG for erythrocytes^41^ and considerably more toxic than PG for mouse blastocysts.^42^ A 20% vol/vol solution of 1,3-butanediol is less toxic than BD for mouse blastocysts.^42^

BD nominally has four stereoisomers,^43^ but two are identical meso-isomers that form a hydrate that readily crystallizes and is cytotoxic.^44,45^ The other two stereoisomers are the entantiomers levo-and dextro-2,3-butanediol, which have identical properties apart from their differing rotation of polarized light.^46^ A racemic mixture of the entantiomers containing only 3.1% wt/wt of the meso-isomer is non-toxic to erythrocytes up to 20% wt/wt, but more toxic than PD at 30% wt/wt.^46^ Nonetheless, BD has a much lower minimum concentration needed to vitrify (Cv) than PD.^47^ It would be more appropriate to compare toxicity of BD and PD at their respective Cv values rather than at equal % wt/wt. Expense has limited the use of BD in cryobiology, so attempts have been made to reduce the cost.^48,49^

There is a dose-dependent reduction in rat heart rate for DMSO concentrations above 0.14 M (1% vol/vol).^50^ Irreversible ultrastructural alterations to rat myocardium occur above 1.41 M (10% vol/vol) DMSO at 30°C, and above 2.82 M DMSO at 15°C.^50^ Osmotic stress is believed to be at least partially responsible for these effects.^50^ Another study showed an increase in action potential duration associated with myocardial cell shrinkage for guinea pig heart muscle exposed to 10% DMSO for 30 min at room temperature.^51^ Aside from osmotic effects, direct blocking action on membrane channel proteins by DMSO molecules is a suggested explanation.^51^

Dermal fibroblasts exposed to DMSO in increasing concentrations between 5% to 30% (vol/vol) at 4°C, 25°C, and 37°C for periods of 10, 20, and 30 min showed decreasing viability with increasing concentration, temperature, and exposure time.^52^ Increasing DMSO concentration from 7.5% to 10% reduces the clonogenic potential of peripheral blood progenitor cells.^185^ Hamster fibroblasts exposed for 1 hr to 10% DMSO at 37°C showed undulations in the cell membrane without swelling, 20% DMSO caused water entry and swelling, and 30% DMSO caused plasma membrane blebs that indicate dissociation between the plasma membrane and the cytoskeleton.^53^ Chondrocytes showed decreasing recovery for increasing temperature (4°C, 22°C, 37°C) and for increasing DMSO concentration (7.5%, 22%, 37%, 44%) for increasing time between 0.5 min and 120 min.^54^

At 20°C DMSO increasingly binds to proteins at concentrations above 40%, which can lead to protein unfolding.^55,56^ Considerable irreversible binding of DMSO to protein has been observed at 10°C.^57^ DMSO has been shown to react with both eye lens protein and glutathione.^58^

DMSO has been reported to decrease the firmness and increase the fluidity of cell membranes.^59^ At 77°C, DMSO decreases cell membrane thickness at low concentrations (2.5%–7.5%), causes the formation of transient water pores at intermediate concentrations (10%–20%), and destroys the bilayer structure at higher concentrations (25%–30%).^52^ Although DMSO can stabilize the gel phase of cell membranes,^60^ above concentrations of 40% DMSO causes the gel-phase structure of ceramide bilayers to undergo a phase transition from gel to liquid crystalline.^61^ All of these effects should increase with increasing temperature. Both the hydrophilicity of DMSO and the capacity of DMSO to destabilize protein conformation increase with increasing temperature.^62^

DMSO protects rat hepatocytes from apoptosis at 1% concentration by shifting caspase-9 into the nucleus (where it cannot initiate apoptosis).^63^ In cultured juvenile rat hippocampal cells, DMSO caused apoptosis in a dose-dependent fashion between 0.5% and 1.0% concentration.^64^ In lymphoma cell lines, DMSO has an anti-apoptotic effect in the concentration range between 1% and 2% over 4–6 days,^58^ but DMSO becomes pro-apoptotic at higher concentrations.^65,66^Ames testing of bacteria with 33% DMSO for 10 min showed a 10-fold increase in mutagenicity.^67^

Rat pup cochlear cells showed a dose-dependent increase in apoptosis when exposed to DMSO concentrations between 0.5% and 6% for 24 hr.^68^ Rat retina cells exposed to DMSO concentrations as low as 0.1% vol/vol for 24 hr exhibited apoptosis.^69^ The DMSO inhibited mitochondrial respiration and elevated cystolic calcium.^62^ For several cell types, including fibroblasts, 1% DMSO increased intracellular calcium two- to six-fold within 5 sec.^70^ Increased intracellular calcium can lead to apoptosis.^71^ DMSO can increase osteoclast cell surface area when not used in high concentrations (high concentration induces osteoclast apoptosis).^72^

Individually, GLY, DMSO, PG, and BD caused corneal endothelial cell loss after exposure for 10–15 min at 0–4°C at concentrations insufficient to vitrify.^73^

## Cell Membrane Toxicity

Cell membrane toxicity is a particular kind of specific toxicity, most frequently associated with DMSO. Cell membrane bilayers consist of hydrophilic polar head groups at the outer and inner surfaces, with hydrophobic fatty acid chains in the middle of the membrane. The ability of molecules to permeate cell membranes increases with lipophilicity, but decreases with increasing molecular size or ability to form hydrogen bonds.^38^

The lifetime of a DMSO–water hydrogen bond is several times the lifetime of a water–water hydrogen bond.^74^ The sulfinyl (SO) oxygen of DMSO hydrogen-bonds to water more strongly (about 30 kilojoules/mole) than water molecules hydrogen-bond to each other (about 20 kilojoules/mole).^75^ DMSO binding with water decreases with increasing temperature.^76^

Membranes are more readily “hydrated” by water than by DMSO, and this relative exclusion of DMSO reportedly causes stress at the membrane interface.^60^ DMSO hydrophobicity and concentration in the lipid bilayer decreases with increasing temperature, which may help explain the increasing toxicity of DMSO with increasing temperature because DMSO localizes around the polar head groups of cell membranes.^60,77,78^ For temperatures above 5°C, addition of FMD to DMSO increases liposome disruption.^76^

A study of erythrocyte hemolysis by alkanols (alkanes having one −OH group), alkanediols (alkanes having two −OH groups), and glycerol (which has three −OH groups) showed that the degree of hemolysis was almost entirely dependent upon the shape change induced in the erythrocytes. A decreasing ratio of solution dielectric constant divided by membrane dielectric constant increased hemolysis. This decreased ratio represented a smaller difference between the hydrophobicity of the membrane and the hydrophobicity of the solution and led to increased membrane surface area exposed to the medium, or membrane vesiculation. Increasing alkanol or alkanediol chain length resulted in increasing hemolysis, whereas addition of a hydroxyl group to an alkanol to produce an alkanediol reduced hemolysis compared to the corresponding alkanol. CPA concentrations that produced 100% hemolysis at 20°C only produced 5%–10% hemolysis at 4°C. On the basis of these results, the authors speculated that combining DMSO (dielectric constant less than that of water) with FMD (dielectric constant greater than that of water) could mutually reduce the toxicity of the two CPAs due to opposing effects of the two CPAs on solution hydrophobicity.^79^ When used intravenously, DMSO has been shown to cause hemolysis.^80^ Of the commonly used CPAs, only FMD has a dielectric constant greater than water.

## Oxidative Damage Due To CPAs

DMSO, METH, GLY, and EG all have anti-oxidant capability, with DMSO being the most potent and GLY the least.^81,82^ But DMSO can be a pro-oxidant by oxidizing free thiol groups on proteins (affecting protein function),^58,83,84^ a reaction that would be expected to decrease at lower temperature.

Plant Vitrification Solution 2 (PVS2) contains 30% GLY, 15% EG, and 15% DMSO.^85^ Shoot tips treated with PVS2 showed lipid peroxidation that could be reduced with melatonin^86^ or vitamins C and E.^87^ Membrane lipid peroxidation in seedlings treated with PVS2 was reduced by glutathione and ascorbic acid.^88^

Cryopreservation of pig ovaries with a vitrification solution containing EG produced oxidative damage that was reduced by anti-oxidant treatment.^89^ Anti-oxidants have been shown to reduce the toxic effects of kidney epithelial cells exposed to oxalate and calcium oxalate.^90^ *N*-acetylcysteine has been shown to reduce glycerol-induced oxidative stress in the kidney.^22^

## Osmotic Damage, Cold Shock, and Chilling Injury

Osmotic damage, cold shock, and chilling injury, unlike oxidative damage, cannot be regarded as a form of CPA toxicity. But these forms of damage can be mistaken for CPA toxicity. CPAs with low permeability can cause more osmotic stress than CPAs with high permeability. Membrane permeabilities of a variety of non-electrolytes, including CPAs, have been studied on a number of cell types, including human blood cells.^38^ Critical factors determining membrane permeability are lipid solubility of the substance (which increases permeability) and hydrogen bonding (which decreases permeability). In general, permeability decreases as the molecular size of the substance increases. In contrast to human blood cells, which are about twice as permeable for DMSO than for GLY, human sperm is nearly three times more permeable for GLY than for DMSO.^91^ For both human red blood cells and sperm cells, permeability to EG is very high compared to the other commonly used CPAs. Yet for mature human oocytes PG has the highest permeability of the most commonly used oocyte CPAs, and EG has the lowest permeability (Table 1).^92^

**Table 1.** Membrane Permeability Coefficient Times 10^−5^ cm/sec for Human Red Blood Cells, Human Sperm, and Human Oocytes

*Cryoprotectant*	*Red blood cells@4°C*^38^	*Sperm @22°C*^91^	*Oocytes @22°C*^92^
Methanol	11.35		
Formamide	8.05		
Ethylene glycol	3.38	13.2	1.95
Propylene glycol	1.79	3.83	3.83
Dimethyl sulfoxide	1.30	1.33	2.60
Glycerol	0.58	3.50	Low

For a variety of cell types, DMSO has many times the membrane permeability of GLY.^93^ EG has about half the permeability of PG or DMSO for human oocytes (and thus increased membrane damage from osmotic stress), but EG is the preferred CPA because it is less toxic.^94^ For pig oocytes, cryopreservation with PG resulted in higher survival than with EG due to greater permeability (and less osmotic membrane damage); but developmental competence of oocytes that survived cryopreservation was greater for EG, suggesting that PG is more toxic.^95^ Human sperm cryopreserved with 1 M EG showed more viability than sperm cryopreserved with 1 M GLY, reportedly because EG is four times more membrane permeable and thus causes less osmotic damage.^91^ However, 2 M EG did not result in better motility than 1 M GLY, possibly due to EG toxicity.^91^ For flounder embryos, EG causes much less osmotic stress than METH, but is much more toxic.^96^ Using survival to hatching as the toxicity assay for flounder embryos exposed to CPAs for 60 min at−15°C resulted in the following order of CPA toxicities, with EG being the most toxic: EG > glycerol > DMSO > METH > PG.^96^ But combining 20% METH with 5% of any of the other CPAs resulted in much less toxicity than combining 20% PG, EG, or DMSO with 5% of any of the other CPAs (except METH).^96^

Although the permeation rate in pig articular cartilage declines in an Arrhenius fashion with temperature for CPAs, the rate of decline nonetheless varies significantly depending on the CPA. Whereas diffusion rate for DMSO declines 25% from 0°C down to −10°C, there is a 50% diffusion rate decline for PG and GLY over the same temperature range.^97^

Testing exposure times and allowing enough permeation time for osmotic stress to be avoided in rabbit kidney tissue slices led to the conclusion that osmotic stress is not the major cause of CPA toxicity for the methods and preparations of those experiments.^98^ But osmotic damage is often associated with cryopreservation of other cells or tissues. Excessive osmotic stress can interfere with protein structure and reduce enzyme activity while causing DNA damage and apoptotic cell death.^99^

Vascular endothelial cells exposed to BD, PG, DMSO, and EG at their vitrifying concentrations at 2–4°C for 9 min showed significantly higher survival for BD or PG than for DMSO or EG. Permeability is highest for BD (4.1), followed by PG (3.0), and then DMSO (2.4) and EG (2.0) (all units in cm/sec × 10^−6^). Raising the temperature from 2–4°C to 22°C increased the permeability 17-fold for BD and DMSO, but only 13-fold for PD and nine-fold for EG. BD is much more toxic than PD at equivalent concentrations, but at the concentrations required to vitrify (32% wt/wt for BD and 45% wt/wt for PG) BD was reportedly less toxic.^47^ (The concentrations to vitrify stated in this paper were reportedly incorrect.^100^) The paper did not suggest whether permeability was a factor in cell survival. Permeability to CPAs such as GLY can vary considerably according to cell type: GLY is highly permeable for human red blood cells, but has a very low permeability for bovine red blood cells.^47^

Chilling injury refers to damage induced in cells held at critical temperatures below temperatures at which cells normally function, whereas cold shock refers to reduced viability due to either a rapid or large decrease in temperature. There is some overlap in the effect on cellular organelles of cold shock and chilling injury, especially in cell membranes. There is also some confusion in the terminology used, at least partially associated with the lack of clarity concerning the mechanisms.

Cold shock most immediately impacts membrane-bound lipids, protein conformation, and nucleic acid conformation. Cold shock inhibits mRNA translation, cold-shock proteins are induced, and there is increased synthesis of more unsaturated fatty acids to increase membrane fluidity.^101^ Initial mRNA translation appears to be the key control point for the cold-shock response in mammalian cells, and oxidative damage can be involved.^102^ Membrane-bound enzyme activity is inhibited, and diffusion rates are reduced. Cold shock proteins may recruit mRNA and ribosomes to the cytoskeleton for translation.^103^

One mechanism of chilling injury in animal cells is probably due to phase transitions in cell membranes.^104^ Lipids in cell membranes would be expected to undergo a liquid-to-gel phase transition in a range between 0°C and 20°C, the temperature range of maximum chilling injury. Chilling sensitivity has been reduced in plants by introducing double bonds into the fatty acids of cell membranes though genetic manipulation^105^ and in sheep oocytes by feeding unsaturated fatty acids to ewes.^106^ Platelets are exceptionally vulnerable to chilling injury and have served as models for cold-induced dysfunction.^107^

Chilling injury increases with exposure time at critical temperatures and, in fact, rapid cooling through the critical temperature range can be a means to reduce chilling injury.^108^ Fish embryos, which are vulnerable to both chilling sensitivity and cold shock, cannot be cryopreserved by such rapid cooling.^109^ Methanol protects zebrafish embryos from chilling injury, a benefit speculated to be due to possible depression of phase transition temperatures in the lipid membranes.^110^ Microtubule polymerization in oocytes is very temperature sensitive, and complete microtubule de-polymerization can occur just above 0°C.^111^ In some cases, re-polymerization of the meiotic spindle occurs on rewarming,^112^ but in other cases irregular chromosomal configurations and abnormal tubulin organization remain after rewarming.^113^

Although chilling sensitivity has been reduced in plants by increasing the degree of fatty acid unsaturation,^105^ much of the chilling injury (or cold shock) in plants has been attributed to free radical damage.^114^ Evidence for free radical damage during chilling has also been seen in houseflies^115^ and sperm^116^ (sperm membranes have a high polyunsaturated fatty acid content).

Cooling experiments of rabbit kidney cortical slices in vitrification solution have been conducted in which viability (potassium to sodium [K^+^/Na^+^] ratios) was used as an index of chilling injury (or cold shock) and have indicated a linear increase in (presumed) chilling injury from 0°C to −85°C.^117^ Gene expression analysis showing “chilling injury” during slow cooling down to −80°C indicated induction of genes related to stress and inflammation.^117^ Hypertonic solutions in the range of 1.2–1.5 times isotonicity completely abolished chilling injury between 0°C to −22°C.^118^ Chilling injury down to −135°C was minimized (85%–90% viability) by cooling to −22°C at 1.2 × hypertonicity and further cooling to −135°C with 1.5 × hypertonicity.^118^ Evidently, some cell shrinkage protects against chilling injury.

## Comparative CPA Toxicities

Chinese hamster ovary cell lines were assayed for chromosome damage after exposure to DMSO, PG, and EG. No chromosome damage was seen for DMSO or EG, but substantial chromosome damage was seen for PG. When a cytochrome P450 oxidation system was added, EG, but not DMSO, showed substantial chromosome damage.^119^ Cytochrome 450 has been shown to metabolize EG to formaldehyde in rats.^120^ In mouse oocyte cryopreservation mixtures, PG caused significant DNA fragmentation, whereas EG and DMSO did not^121^; 99% DMSO, FMD, or METH dissolves DNA.^122^

A study of mouse oocytes comparing EG with DMSO found that both CPAs increased intracellular calcium, but only for DMSO was there an intracellular calcium source.^123^ A different study comparing CPAs for mouse oocyte cryopreservation, PG, DMSO, and EG all increased intracellular calcium content, with PG increasing calcium the most, and EG increasing calcium the least.^124^ The source of the calcium for PG and EG was extracellular, whereas for the DMSO the source was intracellular^124^ (as with the first study).

DMSO, PG, and GLY all showed increasing formaldehyde concentrations as a function of increasing molarity in mouse oocytes, but the formaldehyde increase for PG was more than 30-fold greater than for DMSO or GLY.^125^ Removal of the formaldehyde reduced the zona pellucida hardening. Although the mechanism of formaldehyde production is unknown, the authors suggest it is due to a non-enzymatic reaction in the CPA and solvent.^125^

The minimum concentrations of CPAs that resulted in significant reduction of mouse morula survival with 5 min of 25°C exposure were EG (7 M), GLY (6 M), DMSO (5 M), and PG (4 M).^126^ METH was the second least toxic CPA after EG, showing reduced mouse morula survival after 10 min of 6 M exposure.^126^

For mouse blastocysts exposed to 30% vol/vol CPAs for 10 min at room temperature, EG was by far the least toxic (74.6% of blastocysts subsequently developed) compared to DMSO (25.0%), GLY (21.9%), BD (8.7%), PG (2.1%), or 1,3-butanediol (1.7%).^42^ For mouse blastocysts exposed to 20% vol/vol CPAs for 40 min at room temperature, DMSO was the least toxic (96.7% survived), followed by EG (95.8%), PG (87.5%), GLY (81.7%), 1,3-butanediol (64.2%), and BD (8.3%).^42^

For mouse beta cells, DMSO was found to be more toxic than PG for a range of concentrations and a range of temperatures above 0°C.^127^ On the basis of tests of two cell types, the authors concluded that CPA toxicity is greater for cells with higher metabolic activity.^127^ The toxicities of EG and DMSO for endothelial cells were much greater than the toxicities of PD and BD. Reducing BD concentration from 3.0 M to 2.0 M cut endothelial cell loss by a factor of 35, whereas the same molar reduction of DMSO only cut endothelial cell loss by a factor of 3.^73^

BD was determined to be less toxic to vascular endothelial cells than DMSO, PG, or EG.^128^ In another experiment, the same research team found that exposure of vascular endothelial cells to vitrifying concentrations of BD (2,3 butanediol) (32%), PG (45%), DMSO (45%), and EG (45%) at 2–4°C for 9 min resulted in the greatest cell survival with BD and the least survival with EG. Quantitatively these rates were BD (76.3% survival), PG (63.6%), DMSO (37.0%), and EG (33.2%),^47^ but the team stated that the concentrations needed to vitrify were 32% for BD and 45% for DMSO, EG, and PG, which is reportedly incorrect.^100^ Comparing toxicities of CPAs should be done at Cv for the CPAs rather than for equal % concentrations of the CPAs.

## The qv* Hypothesis of CPA Toxicity

Gregory Fahy has long been studying CPA toxicity and toxicity neutralization.^3,7,8,129^ Fahy and colleagues at 21st Century Medicine, Inc. (21CM) have devised a theory of CPA toxicity based on K^+^/Na^+^ assays of rabbit kidney slices, an ambitious attempt to establish a general theory of CPA toxicity.

Concentration of Na^+^ outside of a cell is typically 10 times that found inside a cell, whereas K^+^ concentration inside a cell is typically 20–35 times greater than outside. The membrane enzyme Na/K-ATPase (the “sodium pump”) uses one molecule of ATP to eject 3 Na^+^ ions in exchange for 2 K^+^ ions brought into a cell.^130^ If the cell membrane is ruptured, or if a cell dies or is no longer able to produce ATP, the normal intracellular K^+^/Na^+^ ratio will be altered. Cell viability can thus be assayed by washing away extracellular material, lysing cells in the sample, and determining the relative concentrations of K^+^ and Na^+^.^57^

Fahy and his colleagues have created a metric denoted as “qv*” which is proposed to measure the average hydrogen-bonding strength between CPA polar groups and water molecules in a solution. Quantitatively, qv* represents the number of moles of water per unit volume divided by the number of moles of polar groups on the CPA at the minimum concentration needed to vitrify under standardized conditions^8^: q = M_W_ (moles water)/M_PG_ (moles polar groups), for v (minimum concentration needed to vitrify, Cv) under standardized conditions (*). The lower the minimum concentration needed to vitrify (v), the stronger the CPA will be.

Polar groups are defined as S
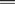
O, C
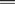
O, OH, and NH_2_, which are more accurately described as hydrogen-bonding groups. The first two groups (S
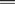
O and C
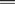
O) will hydrogen-bond with a water hydrogen, whereas hydrogens in the second two groups (OH and NH_2_) will hydrogen-bond with a water oxygen. Of course hydrogen bonding will also occur between CPAs and with other molecules in solution.

A plot of qv* against the viability measure (K^+^/Na^+^ ratio) indicates declining viability with increasing qv*. Thus, qv* is a measure of the vitrifying power of CPA solutions, and is inversely correlated with toxicity.

**FIG. 1.**
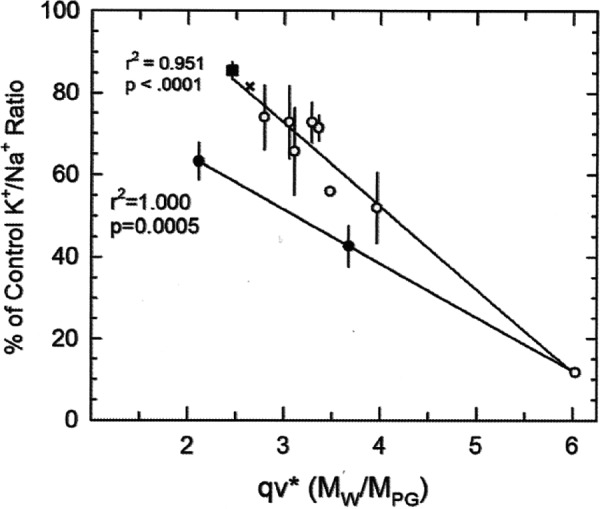
The upper line includes the points most strongly supporting the qv* hypothesis, whereas the lower line includes only dimethylsulfoxide (DMSO) and two solutions described as “outliers,” a line that more weakly supports the qv* hypothesis. Reprinted with permission; Fahy 2010.^129^

All of the toxicity measured on the vertical axis of Fig. 1 is interpreted by Fahy to be non-specific.^8^ Fahy asserts that Fig. 1 demonstrates that a higher qv* results in greater non-specific toxicity, and he interprets the non-specific toxicity to be due to fewer water molecules being available to hydrate macromolecules protectively. Thus, a CPA solution with a higher qv* will be more toxic because fewer polar groups are hydrogen bonding with more water molecules. A CPA that can vitrify with a lower qv* will be less toxic. EG is preferred over PG because EG has weaker average hydrogen-bonding strength per polar molecule, less non-specific toxicity, and leaves more water molecules available to hydrate macromolecules at Cv for EG. (But, EG is described by Fahy as being an outlier due to specific toxicities [Solution 11, Fahy, 2004].)^8^

Two examples are given for calculating qv*. Cv of glucose in water has been determined to be 84% wt/vol^131^ at a cooling rate of 10°C/min (the standardized condition for this experiment, designated “*”). Thus, qv* can be calculated as follows:

\begin{align*} & {840 \ { \rm grams \ glucose / liter} \over 1.303 \ { \rm grams \ glucose /
mL}} \\ & \quad = 645 \ { \rm mL \ glucose \ liter} \Rightarrow
1000 - 645
\\ &  \quad = 355 \ { \rm mL \ H_2O / liter} \ ( 1 \ {\rm gram} = 1 \
{ \rm mL \ H_2O} )\end{align*}

\begin{align*}& 355 \ { \rm gm \ H_2O / liter \,
\times \, mole / 18.0 \ gm \ H_2O} \\ &  \quad = 19.7 \ { \rm mole
\ H_2O / liter} = { \rm M_w} ( { \rm moles \ water} )\end{align*}

\begin{align*}& 840 \ { \rm gm \ glucose / liter
\, \times \, mole / 180 \ gm \ glucose} \\ &  \quad = 4.67 \ { \rm
mole \ glucose / liter} \end{align*}

\begin{align*}& {4.67 \ { \rm mole \ glucose \, \times \, 6} \ {
\rm polar \ groups} = 28.0} \\ &  \quad = { \rm M_{PG}} \ ( { \rm
moles \ polar \ groups} )\end{align*}

\begin{align*} { \rm qv^* } = \frac { { \rm M_w } \ ( { \rm moles
\ water } ) }  { { \rm M_ { PG } } \ ( { \rm moles \ polar \
groups } ) } = 19.7 / 28.0 = 0.704\end{align*}

Projecting the upper line in Fig. 1 backward indicates that any qv* value below about 1.5 will correspond to 100% viability. So the qv* value of 0.704 indicates that glucose should have no non-specific toxicity.

For the second example, qv* is calculated for the DMSO plus polyvinylpyrrolidone K30 (PVP) solution (solution 1 of Fahy 2004^8^) using the standardized conditions described in Table 1 of the paper positing the qv* hypothesis.^8^ Solution 1 includes 6% PVP and 2 mL/100 mL carrier solution for 100 MPa pressure (about 1000 atmospheres) and a cooling rate of 10°C/min (the standardized condition for this experiment,*). The high pressure and the presence of PVP means that Cv of DMSO will be less than the 49% wt/vol indicated in Table 2 of this article. Under these conditions, the Cv of the total solution (DMSO + PVP) is 47% wt/vol and the Cv of DMSO is 47 − 6 = 41% wt/vol. Polar groups for PVP were not counted, because PVP is not a permeating CPA, despite the fact that PVP reduces the Cv for DMSO in the solution.

\begin{align*}& 1000\ \rm {mL \ of \ the\ DMSO} \\ &  \quad  + \rm
{PVP \ solution \ has \ a \ mass \ of \ 1077.5 \ grams}
\end{align*}

\begin{align*}& 1077.5 \ {\rm grams} - ( 410 \ {\rm grams \ DMSO} +
\, 60\rm \ grams \\ &  \quad  {\rm PVP} + {\rm 39 \ grams \ RPS \
- 2 \ carrier \ solution} ) \\ &  \quad  = 568.5 \ {\rm grams \
H_2O / liter}
\end{align*}

\begin{align*} & 568.5 \ { \rm grams \ H_2O / liter
\, \times \, mole / 18.0 \ grams \ H_2O} \\ &  \quad = 31.6 \ {
\rm mole \ H_2O / liter} = { \rm M_w} \ ( { \rm moles \ water}
)\end{align*}

\begin{align*}& 410 \ { \rm grams \ DMSO / liter
\, \times \, mole / 78.1 \ grams \ DMSO} \\ &  \quad = 5.25 \ {
\rm mole \ DMSO /liter} \end{align*}

\begin{align*}& 5.25 \ { \rm mole \
DMSO \, \times \, 1 \ polar \ groups} = 5.25 \\ & \quad  = { \rm
M_{PG}} \ ( { \rm moles \ polar \ groups} )\end{align*}

\begin{align*} { \rm qv^* } = \frac { { \rm M_w } \ ( { \rm moles
\ water } ) }  { { \rm M_ { PG } } \ ( { \rm moles \ polar \
groups } ) } = 31.6 / 5.25 = 6.02 \end{align*}

**Table 2.** Concentration Needed to Vitrify (Cv) for Selected Penetrating Cryoprotectants at One Atmosphere Pressure

*CPA*	*Cv %wt/vol*
PG	43.5
DMSO	49–50
EG	55
GLY	65

Values for all CPAs taken from Fahy 1984^100^ and include carrier solutions of 2 mL/100-mL solution.

CPA, cryoprotectant; PG, propylene glycol; DMSO, dimethylsulfoxide; EG, ethylene glycol; GLY, glycerol.

Both 1,2-propanediol and 1,3-propanediol have two polar OH groups, but compared to the 1,2-propanediol molecule, 1,3-propanediol has a higher concentration needed to vitrify (57% versus 44%).^132^ The qv* hypothesis thus correctly predicts that PG will be more toxic.

## Some Questions About the Qv* Hypothesis

The polar groups S
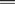
O, C
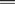
O, OH, and NH_2_ do not have equal hydrogen-bonding strength, and the hydrogen-bonding strength of each of those groups will vary over a range depending on the hydrogen-bonding partner. For example, the OH group on METH hydrogen-bonds to water nearly 20% more strongly than hydrogen bonding between water molecules.^31^ NH_2_ is counted as being a single polar group, despite the possibility that NH_2_ could present two hydrogen atoms for hydrogen bonding. Similarly, only the NH_2_ and C
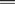
O groups on FMD are counted as polar groups (two polar groups), despite the oxygen, nitrogen, and three hydrogen atoms potentially available for hydrogen bonding. Other researchers have suggested that a hydroxyl group can form two hydrogen bonds, whereas an amide group can form three.^133^ Intra-molecular hydrogen bonding increases as hydroxyl groups move closer together.^133^ In his 2010 paper on toxicity neutralization, Fahy commented that either only one NH_2_ in urea can contribute to toxicity, or that the overall polarity of the urea molecule is more important for toxicity than individual polar groups.^129^ This comment conflicts with the justification for counting polar groups to calculate qv*.

Hydrogen bonds are much stronger in a non-polar environment than in a polar microenvironment.^134^ Hydrogen bonding between water molecules can become up to six-fold stronger in an acid or basic medium.^135^ Adding a methyl group to an alcohol, as essentially happens in going from EG to PG, results in increasing the hydrogen-bonding strength of the oxygens on the hydroxyl groups,^132^ thus reducing Cv while increasing toxicity.

In positing the qv* hypothesis, the authors state: “qv* is a measure of the glass-forming efficiency of the CPAs that compose the vitrification solution.” ^8^ But Cv measures glass-forming efficacy, so treating each polar group as making an equal contribution to qv* when they are in fact not equal must introduce imprecision, despite the fact that qv* is intended to determine the average hydrogen-bonding strengths of all the counted polar groups.

Precise determination of the hydrogen-bonding strengths of each of the polar groups could conceivably allow for more precise qv* type calculations. Hydrogen bond strengths often cannot be determined directly, which is why hydrogen bond experts prefer using hydrogen bond lengths as a more easily measured surrogate for hydrogen bond strength.^136^ The length of a hydroxyl hydrogen hydrogen-bonded to an oxygen is slightly shorter (stronger) than the length of an amine hydrogen hydrogen-bonded to an oxygen.^136^

By the qv* hypothesis, toxicity and vitrification effectiveness are the result of the same process: Hydrogen bonding of CPAs to water molecules, although a vitrification solution that requires more polar groups per water molecule to vitrify will be less toxic. Vitrification can occur either by strong hydrogen bonding of water molecules with a CPA having a lower concentration needed to vitrify (like PG), or by weak hydrogen bonding of water with a CPA requiring a higher concentration to vitrify (like EG). According to the qv* hypothesis, the weaker hydrogen-bonding CPA will be less toxic because more water molecules will be available to hydrate macromolecules.^8^ Although more water molecules will also be available to form ice, the colligative “dilution” of water by the weak CPA should prevent ice formation. It seems contradictory that water remains available for hydration, but not available for ice formation. By definition, water molecules that hydrate macromolecules are bound to those macromolecules (“bound water”). Only water molecules not bound to macromolecules (“unbound water”) are capable of ice formation. If CPAs hydrogen-bond to both “bound water” and “unbound water,” then it could be possible that less hydrogen bonding by CPAs could allow more “bound water” to hydrate macromolecules.

The K^+^/Na^+^ assay measures toxicity, but does not distinguish between specific and non-specific toxicity. Viability could be reduced by many specific toxicities other than lack of macromolecule hydration that results from the binding of CPAs to water attributed to non-specific toxicity. Although Fahy reported that 30% wt/vol DMSO has little effect on K^+^/Na^+^,^7^ ample evidence has been given at the beginning of this reveiw about the specific toxicities of DMSO at concentrations below the 41% w/v that is the basis of the calculated value of qv* for DMSO. And combining DMSO with the non-penetrating CPA PVP (without counting polar groups on PVP) to reduce DMSO Cv from 47% to 41% seems dubious (although all the penetrating CPAs used by Fahy to determine qv* had non-penetrating CPAs).

There are three notably extreme points in the plot of viability (K^+^/Na^+^) versus qv* (Fig. 1) demonstrating a relationship between the two variables for a variety of CPAs.^8^ The first most extreme, DMSO with a qv* value of 6.02 (described by Fahy as an “anchor point”), more than any other point defines the graph and justifies the qv* hypothesis. The other two extreme points (along with DMSO) define a line below the qv* hypothesis line. EG with a qv* value of 2.11 (solution number 11 of Table 1 of the 2004 paper defining qv*^8^) is described as being an outlier due to some unexplained specific toxicity,^8^ although the same explanation cannot be given for solution number 7. Hydrogen-bonding strength of CPAs to water weakens as CPA concentration increases.^137^ The qv* metric does not account for this fact.

## Toxicity Reduced by Combining CPAs

FMD is reputedly the most toxic CPA,^129^ but FMD does not bind to water strongly^138^ and cannot vitrify on its own. FMD presumably has specific rather than non-specific toxicities. For years, Fahy believed that FMD reduces the toxicity of DMSO but not vice versa; however, in 1990, he found evidence that FMD does not reduce DMSO toxicity.^7^ In 1995, he found evidence that DMSO reduces the toxicity of FMD.^139^ DMSO reportedly does not hydrogen-bond with FMD at 4°C, and the two molecules evidently repel each other in aqueous solution.^139^ Because FMD more strongly self-associates than associates with DMSO or water, what appears to be a reduction of DMSO toxicity by FMD in aqueous solution could simply be a dilution effect. Acetamide has remarkably low toxicity for kidney slices, but is less amenable than FMD to toxicity neutralization by DMSO.^129^ The mechanism of DMSO reduction of FMD toxicity remains unexplained.

A study of the effect of CPA toxicity on the viability of pig articular chondrocytes at 37°C found PG to be the most toxic, DMSO and FMD to be somewhat less toxic, whereas EG and GLY to be the least toxic. Viability was assayed on the basis of both membrane integrity and metabolic activity. Care was taken to avoid osmotic damage by using no greater than a 3 M solution concentration. Maximum exposure time was 120 min. All CPA combinations showed toxicity reduction, with PG or FMD combined with DMSO both showing equivalent toxicity as assessed by cell viability. The combination of DMSO with FMD showed greatly reduced toxicity below that of either DMSO or FMD alone.^140^ This result was interpreted to indicate that both DMSO reduces FMD toxicity and FMD reduces DMSO toxicity. As stated in the previous paragraph, Fahy's interpretation would be that the apparent reduction of DMSO toxicity by FMD is by dilution rather than by toxicity neutralization. The authors concluded that all two-CPA combinations were less toxic than single CPAs at the same final concentration. A 3 M solution of EG-DMSO-GLY best preserved membrane integrity, whereas 3 M EG best preserved metabolic activity.

When the team used human chondrocytes to study the toxicity of 6 M and 8.1 M solutions of DMSO, EG, FMD, GLY, and PG at 37°C, they found that all the three-CPA combinations they examined had interactions that reduced toxicity. Two-CPA and four-CPA combinations, by contrast, had increased toxicity. The toxicity of the two-CPA combinations was attributed to CPA–CPA interactions. Why human rather than pig chondrocytes or why increased CPA concentration would result in different results for two-CPA combinations was not explained, although the results were not strictly comparable because the CPA concentrations differed between the experiments. Combining PG with any of the other CPAs resulted in greater toxicity compared to the other combinations. The authors speculated that PG might polarize the molecular charges of the other CPAs, making them more toxic.^141^

The combination of DMSO and EG showed reduced toxicity for buffalo oocytes.^14^ Mouse oocytes exposed to 1.5 M solutions of DMSO, PG, and EG at 23°C for 15 min showed considerably greater survival for DMSO and EG than for PG. But combining DMSO and PG considerably reduced the toxicity of both CPAs, thereby increasing cell survival.^143^ Mouse blastocysts have been successfully cryopreserved using a vitrification solution composed of EG, DMSO, and 1,3-butanediol.^142^ Hemolysis from BD containing 3.1% wt/wt of meso-isomer at 4°C was drastically reduced by adding 4% wt/wt of trehalose, sucrose, sorbitol, or mannitol.^144^ Trehalose and sucrose reduced hemolysis more effectively than sorbitol or mannitol.

Understanding the means by which combining CPAs reduces CPA toxicity could be a way of understanding the mechanisms of CPA toxicity as well as a way of finding better combinations. The cell or tissue-specific effects are important for that understanding.

## Cryopreservation Minimizing CPA Toxicity

Insofar as CPA toxicity is the key factor limiting vitrification, an investigation of the subject of CPA toxicity should ideally be directed at finding means to minimize that toxicity. To the extent that oxidative damage, osmotic damage, cold shock, or chilling injury are implicated in damage that could be mistakenly attributed to CPA toxicity, efforts should be made to decisively identify the cause of damage.

As described in the previous section, mixtures of CPAs can be less toxic than individual CPAs. This fact could raise suspicion about the existence of a concept of “non-specific toxicity” common to all CPAs as a consequence of CPA hydrogen bonding of water. Toxicity neutralization could be a dilution effect if all CPA toxicity were specific. Understanding the mechanisms of toxicity neutralization could be an important step toward discovering the mechanisms of CPA toxicity and toward discovering better means of reducing CPA toxicity.

High pressure will reduce the concentration of a CPA needed to vitrify (Cv), thereby reducing the toxicity of the vitrification solution required. At 200 MPa pressure, the homogeneous nucleation temperature of water decreases from −40°C to −92°C, such that even pure water can be vitrified at reasonably achievable cooling rates.^145^ Pressures below 100 MPa are less damaging to tissues than pressures above 100 MPa.^100^ Such pressures have formerly been used in cryopreservation, but this practice is rarely used currently.^100^

The CPA polar groups S
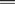
O, C
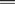
O, OH, and NH_2_ of the qv* hypothesis are not the only polar groups in a solvent that can hydrogen bond to water. Like CPAs, kosmotropic (order-making) co-solvents bind to water molecules and act to modify water structure by forming hydrogen bonds.^146,147^ Kosmotropic co-solvents can be ionic (such as carbonate, sulfate, and Mg^2+^) or non-ionic (such as polyhydroxyl compounds like sugars). Kosmotropic anions have high charge density, are very polarizable, interact more strongly with water than water interacts with itself, and compete with the water molecules associated with protein surfaces (water that hydrates proteins).^147^ Kosmotropic co-solvents enhance the stability of proteins by being preferentially excluded from the solvation shell of the proteins.^147,148^ DNA is stabilized by the kosmotropic substance glucose, whereas urea can be a hydrophobic chaotropic co-solvent causing protein denaturation.^149,150^

But although kosmotropic co-solvents have the same water-binding and dehydrating effect as CPAs reputedly have, kosmotropes are demonstrably protective rather than toxic. Kosmotropic protectiveness is apparently due to the same properties attributed to toxicity under the qv* hypothesis. Kosmotropes do not vitrify, but perhaps like FMD (which does not vitrify on its own) kosmotropes could assist vitrification. Ca^2+^ and Mg^2+^ have been shown to increase the glass transition temperature of glycerol,^151^ and HPO_4_^2−^ has been shown to increase the glass transition temperature of trehalose.^152^ As with the comparison of EG and PG, kosmotropes could assist CPA vitrification by weak hydrogen bonding, leaving more water available for hydration. Inadvertent kosmotropic enhancement may already be implemented by the use of carrier solutions, which can reduce CPA toxicity without reducing CPA concentration.^57,153,154^ With knowledge of the mechanisms, carrier solutions could be selected that minimize CPA toxicity. Insofar as CPAs become less toxic with cooling and will have differing degrees of toxicity depending on the CPA, more toxic CPAs (or higher concentrations of CPAs) can be introduced at lower temperature to reduce CPA toxicity.^2^

Penetrating CPAs (CPAs that cross cell membranes and enter cells) are often used with non-penetrating CPAs (which do not enter cells) because ice more readily forms extracellularly than intracellularly.^155^ With non-penetrating CPAs preventing ice formation, penetrating CPA solutions need not be so concentrated (or toxic). Ice-blocking agents are a special class of non-penetrating molecules that can assist vitrification by reducing ice formation. These include polyvinyl alcohol^156^ and polyglycerol.^157^

The penetrating CPAs discussed in this paper (BD, DMSO, EG, FMD, GLY, METH, and PG) are those most commonly used in cryobiology, but other penetrating CPAs can be used, including urea,^129^ acetamide,^129^ *N*-methylformamide,^129^ *N*,*N*-dimethylformamide,^129^ diethylene glycol,^129^ triethylene glycol,^158^ *n*-propanol,^159^ isopropanol,^159^ 1,3-propanediol,^132^ 1,3-butanediol,^42^ 2-methoxyethanol,^160^ and 3-methoxy-1,2-propanediol.^159^ Mass assays of mixtures of various CPA combinations might find combinations that have low toxicity for various cell and tissue types.

Replacement of hydroxyl groups by methoxyl groups can result in compounds that are less viscous, interact more with water (less self-interaction), vitrify at higher temperatures, reduce critical cooling rate by at least an order of magnitude, and penetrate cell membranes more readily.^160^ Self-interaction of hydroxyl groups on CPAs reduces the interaction of CPAs with water, an effect not seen with methoxyl groups insofar as the oxygen on those groups can interact with water, whereas there is no interaction between methoxyl groups. But reducing Cv can result in increasing CPA toxicity, which has been demonstrated in the case of 2-methoxyethanol in the form of membrane damage visible under electron microscope.^161^

Sugars are often used as extracellular CPAs because of their low toxicity. The qv* calculation for glucose in water above suggests no non-specific toxicity for glucose. Northern wood frogs use high concentrations of glucose as a cryoprotective agent, both intracellularly and extracellularly.^162,163^ Glucose does, however, have specific toxicities, such as binding to protein^57^ and as a reducing sugar causing glycation.^164^ A 220 mM d-galactose solution was shown to be nearly as effective a CPA as 5% DMSO for human embryonic liver cells (and substantially better than d-glucose),^165^ but galactose is more glycating than glucose.^166^

Monosaccharides can dissolve in CPA solutions more readily and vitrify at lower concentrations than disaccharides,^167^ but because of their capacity for glycation, monosaccharide exposure to protein should be brief and at low temperature. Sucrose is regarded as a kosmotrope.^148^ Sucrose is used as an extracellular CPA for vitrification of embryos and oocytes.^167^ But in acidic conditions sucrose is far more vulnerable to hydrolysis into its reducing sugar monosaccharides than the disaccharide trehalose.^168^

Trehalose is a soluble, non-reducing disaccharide of glucose molecules. Trehalose is synthesized intracellularly from glucose in organisms that undergo dehydration. Trehalose can replace the “bound” water surrounding macromolecules and protectively “hydrate” those macromolecules by substituting for water.^169^ For many anhydrobiotic organisms, trehalose can constitute up to 20% of dry weight.^170^ Hydrogen-bonding strength is lower for sugars with a higher glass transition temperature.^186^

Loading trehalose into fibroblasts and keratinocytes by reversible permeabilization of cell membranes allowed most of those cells to survive cryopreservation.^171^ Microinjection has been used to get trehalose into human oocytes, which improves cryopreservation.^172^ When combined with 0.5 M DMSO, 0.5 M trehalose microinjected into mouse oocytes resulted in excellent cryosurvival and healthy offspring (presumably because trehalose alone would not enter organelles such as mitochondria and endoplasmic reticulum).^173^ Plasmids containing the trehalose transporter TRET1 from African chironomid larvae have been transfected into Chinese hamster ovary cells, resulting in a seven-fold increase in trehalose uptake.^174^ Gene therapy might allow trehalose or other non-penetrating CPAs to be synthesized within cells.

Artificial ion channels and nanotubes in cell membranes could be a means of getting large non-toxic vitrifying molecules that are normally non-penetrating into cells and tissues for intracellular vitrification.^175–178^ Detergents could be used for the same purpose.^179^ Because hydrogen bonds are much stronger in a nonpolar environment than in a polar microenvironment^134^ and hydrogen bonding between water molecules can become up to six-fold stronger in an acid or basic medium,^135^ it may be possible to control CPA toxicity by adjusting pH or the polarity of the microenvironment.

Transplantable hearts, kidneys, pancreases, and livers can be preserved hypothermically by replacing blood in blood vessels with cold gas rather than cold fluid.^180^ Arigos Biomedical, Inc. has used cold helium gas to cool a pig kidney down to −180°C without fracture, and the company believes that using 20 atmospheres of pressure could allow for 100 times faster cooling rates.^181^ Such rapid cooling rates could reduce CPA exposure time, thereby reducing toxicity provided damage due to cold shock and endothelial cell dehydration can be avoided.

## Reversal of CPA Toxicity

If CPA toxicity during organ, tissue, or cell cryopreservation causes caspases, proteases, or kinases to be released, leading to apoptosis, interventions could be applied to reverse these processes.^182^ Caspase inhibitors have been used to block apoptosis in cryopreserved hematopoietic cells rewarmed from cryogenic temperatures.^183^ Lesser forms of CPA toxicity might be reversed by epigenetic modifications. Gene expression changes associated with chilling injury have been assayed,^117^ and those changes could have been due to epigenetics. Computer-based systems of drug discovery that alter metabolism to a healthy state have been validated,^184^ and such systems might be applied to metabolic dysfunction induced by CPAs.

## Concluding Remarks

Attempts to explain CPA toxicity are urgent and laudable. CPA toxicity should be understood if it is to be reduced by means other than trial and error. To understand CPA toxicity, it is necessary to understand what macromolecules or organelles are being chemically damaged and how they are being damaged. Various cells or tissues should be exposed to various CPAs followed by examination of those cells or tissues for damage to DNA, proteins, mitochondria, etc. Then an effort should be made to determine the molecular mechanisms that caused the damage.

If individual CPAs can neutralize other individual CPAs, the mechanism of this neutralization should be determined. If dehydration damage is the mechanism of non-specific CPA toxicity, this needs to be demonstrated. Electron microscopy could potentially supplement macromolecule and organelle damage assays. Without exact assaying of molecular damage, explanations of CPA toxicity can only be speculation.
